# Exhaled biomarkers in adults with non-productive cough

**DOI:** 10.1186/s12931-023-02341-5

**Published:** 2023-03-01

**Authors:** Össur Ingi Emilsson, Spela Kokelj, Jörgen Östling, Anna-Carin Olin

**Affiliations:** 1 Department of Medical Sciences, Respiratory, Allergy and Sleep Research, Uppsala University, 75185 Uppsala, Sweden; 2 Occupational and Environmental Medicine, School of Public Health and Community Medicine, Institute of Medicine, Sahlgrenska Academy, University of Gothenburg, Gothenburg, Sweden; 3 PExA AB, Gothenburg, Sweden

**Keywords:** Chronic cough, Exhaled biomarkers, PExA

## Abstract

**Background:**

Chronic cough is a common condition but disease mechanisms are not fully understood. Our aim was to study respiratory biomarkers from the small airways in individuals with non-productive cough.

**Methods:**

A cohort of 107 participants answered detailed questionnaires, performed spirometry, exhaled NO measurement, impulse oscillometry, gave blood samples and particles in exhaled air (PEx) samples. Current smokers (N = 38) were excluded. A total of 14 participants reported non-productive cough (cases). A total of 55 participants reported no cough (control group). PEx samples, containing exhaled particles derived from small airways, were collected and analysed with the SOMAscan proteomics platform.

**Results:**

Participants with non-productive cough had similar age, sex, BMI, and inflammation markers in blood tests, as participants without cough. The proteomics analysis found 75 proteins significantly altered among participants with chronic cough compared to controls, after adjusting for sex and investigator performing the PExA measurement (all with p-value < 0.05 and q-value ≤ 0.13, thereof 21 proteins with a q-value < 0.05). These proteins were mostly involved in immune and inflammatory responses, complement and coagulation system, but also tight junction proteins and proteins involved in neuroinflammatory responses.

**Conclusions:**

This exploratory study on proteomics of exhaled particles among individuals with chronic cough found alterations in relative abundance of 75 proteins. The proteins identified are implicated in both pathways known to be implicated in cough, but also potentially new pathways. Further studies are needed to explore the importance of these findings.

## Background

Chronic cough is a common condition in the general population with many possible causes, but often difficult to treat [1]. In recent years, advances have been made in understanding the origin of cough, resulting for example in the identification of the cough hypersensitivity syndrome [2]. Numerous neuroreceptors in the airways have been identified as being able to induce cough [3]. However, drugs that block these receptors have often been unsuccessful, with the exception of P2X3 inhibitors which have shown some promise [4]. A better understanding of the mechanisms responsible for chronic cough and mechanisms responsible for initiating the cough reflex is therefore needed to be able to better treat this debilitating condition.

The sensory nerves initiating the cough reflex have various locations. They are well known to be present in the larynx and proximal airways, but have also been shown to be present in more distal airways [5, 6]. Also, a study performing bronchial provocation test using hypertonic saline on patients with chronic cough found a significant change in small airway function as measured by impulse oscillometry [7]. These findings indicate that small airway disease may associate with chronic cough, even though this is not extensively studied.

Several mechanisms may interact to increase the cough susceptibility in addition to upregulated airway sensory nerves. Epithelial barrier function may be impaired through oxidative stress, which in turn may potentially make the underlying respiratory nerves more susceptible to external stimuli [8–11].

The immune system has also been suggested to play a role in the pathogenesis of chronic cough, through interaction with the nervous system [12]. For example, eosinophils may stimulate vagal C-fibers which are important in the cough reflex. Also, increased concentrations in induced sputum samples of inflammatory biomarkers such as histamine, prostaglandin D2 and E2 have been reported in idiopathic cough, which may be protussive mediators [12–14]. However, as induced sputum mainly stems from the larger airways, it is unknown if the same processes are at play in the distal airways.

Identifying biomarkers that are easy to measure and reflect cough mechanisms would be highly valuable for better evaluation of patients with chronic cough [15]. A novel method for collecting samples of the small airways respiratory tract lining fluid non-invasively has been developed and validated, a method called Particles in Exhaled Air (PExA). PEx samples have never been analysed among individuals with chronic cough.

The aim of this exploratory study was to evaluate the proteomic profile of the small airways, collected non-invasively in the form of exhaled particles, among individuals with chronic cough.

## Methods

A total of 107 subjects were recruited from our previous studies or by an advertisement in a daily paper, and examined in 2016–17. The cohort consisted of 38 current smokers, 47 former smokers and 22 healthy never smokers. Current smokers were excluded from this study. The inclusion criterion for never smoking controls was post-bronchodilation FEV1/FVC > 0.70. Subjects were defined as current smokers if they had smoked on a regular daily basis for at least one year at the time of the examination. Former smokers were defined as those that had not smoked in the last 12 months, but had smoked on a regular daily basis prior to that. Those who had never smoked on a regular daily basis were classified as never smokers.

Participants provided written informed consent prior to the measurements and the Regional Ethics Committee at the University of Gothenburg approved the study (442-17 and 390-06).

All subjects answered a detailed questionnaire on medical history and symptoms, and performed spirometry, impulse oscillometry (IOS) and fractional exhaled nitric oxide (FeNO). Exhaled particles were collected using the PExA method. Blood samples were obtained and analysed for hsCRP and white blood cell differential count.

All subjects were instructed to withdraw from short-acting bronchodilators and long-acting bronchodilators at least 6 h and 24 h prior to the examination, respectively.

As current smoking has been found to have a significant effect on the small airway protein profile [16], current smokers were excluded from further analysis.

### Non-productive cough

Non-productive cough was defined as a positive reply to the question “Have you during the last 12 months had dry cough, i.e. cough without sputum?” Altogether 24 participants answered “Yes” and 83 answered “No”. Of the 24 participants with non-productive cough, 10 were current smokers, and therefore 14 were eligible for the main analysis.

Of the 83 participants without cough, 28 were current smokers, and therefore 55 were eligible for the main analysis.

### Lung function and asthma definition

The participants performed spirometry using the Spirare spirometer (Spirare, Stockholm, Sweden). Reversibility test was performed using 400 µg of salbutamol in accordance with the ATS/ERS criteria [17]. Forced vital capacity (FVC), forced expired volume in one second (FEV1) an FEV1/FVC ratio were expressed as a percentage of the reference value (% pred) according to Brisman et al. (note the corrigendum) [18].

The participants also performed IOS using a Jaeger Masterscreen system (CareFusion, Würzburg, Germany) before and after bronchodilation with 400 µg of salbutamol in accordance with the ERS task force criteria [19]. The mean values of resistance at 5 Hz and 20 Hz (R5 and R20), frequency dependence of resistance (R5-R20) and area under the reactance curve (AX), were calculated and expressed as % predicted according to Kjellberg et al. [20]

Asthma was defined as a positive reply to the question “Do you have a physician-diagnosed asthma?”.

### Particles in exhaled air

Exhaled particles (PEx) were collected using the PExA instrument version 1.0 (PExA AB, Gothenburg, Sweden), as previously described [21, 22]. The measured particle sizes cover diameters between 0.41 and 2.98 µm. Subjects inhaled HEPA-filtered air for a minimum of three breaths before the sampling in order to remove particles from ambient air. All participants wore a nose clip throughout the procedure. A standardized breathing manoeuvre was used [23, 24], starting with an exhalation at normal flow rate to residual volume, breath holding for 5 s, followed by a maximal inhalation to total lung capacity, immediately followed by a normal exhalation to functional residual capacity. Between breathing manoeuvres, the subject breathed particle-free air tidally for 30 to 60 s. Each sampling session continued until 120 ng of exhaled particles were collected. After collection the sample holder was transferred to a clean air room and the substrate was cut out with a scalpel from the sample holder area and placed in Millipore Ultrafree-MC LH Centrifugal Filter insert (FC30LH25) and stored at – 80 °C for subsequent extraction and SOMAscan analysis. True blank samples were generated by applying the same sample handling procedure as for real samples but without collecting PEx sample from the study subjects.

### SOMAscan proteomic analysis and processing of data

The SOMAscan (SomaLogic Inc, Boulder, USA) proteomics platform that uses slow off-rate modified DNA aptamers can simultaneously quantify more than 1300 human proteins in blood samples. SOMAscan analysis of PEx samples and processing of data has been previously described in detail [16]. In short, prior to SOMAscan analysis, the volume of sample buffer was adjusted to reach the same concentration of PEx in all samples in order to normalize the samples for the differences in the collected amount of PEx. Intra-plate and inter-plate normalization were performed by SomaLogic according to their SOMAscan assay good laboratory practice (GLP) data quality-control procedures. Limit of detection (LOD) was calculated as the relative fluorescent unit (RFU) mean plus 3 standard deviations based on two blank samples. For the primary analysis, proteins with (RFU) values > LOD in more than 50% of the samples were considered for further analyses. For a secondary analysis, proteins above the LOD among more than 50% of those with non-productive cough, but among less than 50% of those without cough, were identified.

### Statistical analysis

As mentioned above, active smoking can have a significant effect on the small airway protein profile, and therefore current smokers were excluded from the primary analysis.

Statistical analyses of the protein data were performed using general linear model-based statistics (Qlucore Omics Explorer 3.8 software, Qlucore AB, Lund, Sweden). SOMAscan data was log_2_ transformed before the analysis to achieve normal distribution. General linear model, with each variable normalized to mean 0 and variance of 1, was used to determine differences in protein abundance between subjects with and without non-productive cough. The analysis was adjusted for the investigator performing the PExA measurements, as well as the sex of the subjects.

To identify which biological pathways the identified proteins were mostly involved in through protein–protein interactions, a STRING (Search Tool for the Retrieval of INteracting Genes/proteins) analysis was performed using an online available tool [25].

In keeping with the exploratory nature of this study, protein differences between groups with a p-value < 0.05 were considered to be of interest. A q-value was also calculated using the Benjamini–Hochberg correction for multiple testing adjustment. Statistical analysis of clinical and demographic data was performed using IBM SPSS Statistics for Windows, version 28 (IBM Corp., Armonk, N.Y., USA) with the significance level set to p < 0.05.

## Results

### Cohort characteristics

The participants’ baseline characteristics are presented in Table 1. In summary, participants with non-productive cough had a similar age, BMI, CRP, blood eosinophils, IOS, and FeNO, as participants without cough. Also, spirometry results were largely similar, except for a slightly higher FVC percent predicted among participants with non-productive cough, compared with participants without cough, however the p-value did not reach the level of statistical significance (Table 1). Asthma was present in one of 14 participants with non-productive cough.

**Table 1:** General characteristics and clinical data of the subjects included in the study

	Subjects without chronic non-productive cough (n = 55)	Subjects with chronic non-productive cough (n = 14)	p-value
Age (years)	59 (53–67)	61 (53–67)	0.654
Sex (F/M)	30/25	8/6	0.862
BMI (kg/m^2^)	25.5 (23.7—27.6)	26.4 (24.9–28.7)	0.251
Former smokers, N (%)	36 (65%)	11 (78%)	0.347
Pack-years	15 (0–26)	(18 (3–40)	0.342
Asthma diagnosis, N (%)	2 (4%)	1 (7%)	0.566
hsCRP (mg/L)	0.91 (0.61–1.40)	1.75 (0.52–3.70)	0.180
Eosinophils (10^9^ cells/L)	0.10 (0.10–0.20)	(0.10 (0.08–0.20)	0.505
FeNO (ppb)	17 (13–28)	15 (12–21)	0.396
FVC % pred^a^	95.9 (89.0–100.3)	104.1 (91.2–109.1)	0.054
FEV1% pred^a^	93.4 (85.2–97.2)	97.0 (85.9–105.7)	0.495
FEV1/FVC^a^	76.4 (71.2–79.8)	75.9 (66.1–78.1)	0.249
R5 Hz (kPa/(L/s))^a^	0.330 (0.270–0.390)	0.305 (0.285–0.398)	0.987
R5 Hz % pred^a^	94.0 (83.9–107.3)	93.8 (84.6–105.9)	0.867
R5-R20 (kPa/(L/s))^a^	0.043 (0.025–0.063)	0.022 (0.015–0.072)	0.472
R5-R20% pred^a^	75.8 (45.2–128.8)	42.5 (29.7–129.0)	0.453
AX (kPa/L)^a^	0.213 (0.105–0.328)	0.143 (0.092–0.401)	0.710
AX % pred^a^	83.6 (46.6–145.1)	65.1 (44.8–165.7)	0.685

Data is displayed as median and interquartile range (IQR), unless specified otherwise. P-values are based on a nonparametric Mann-Wthitney test for continuous data and Chi-square test for categorical data

^a^post-bronchodilation

### Proteomics results

**Fig. 1:**
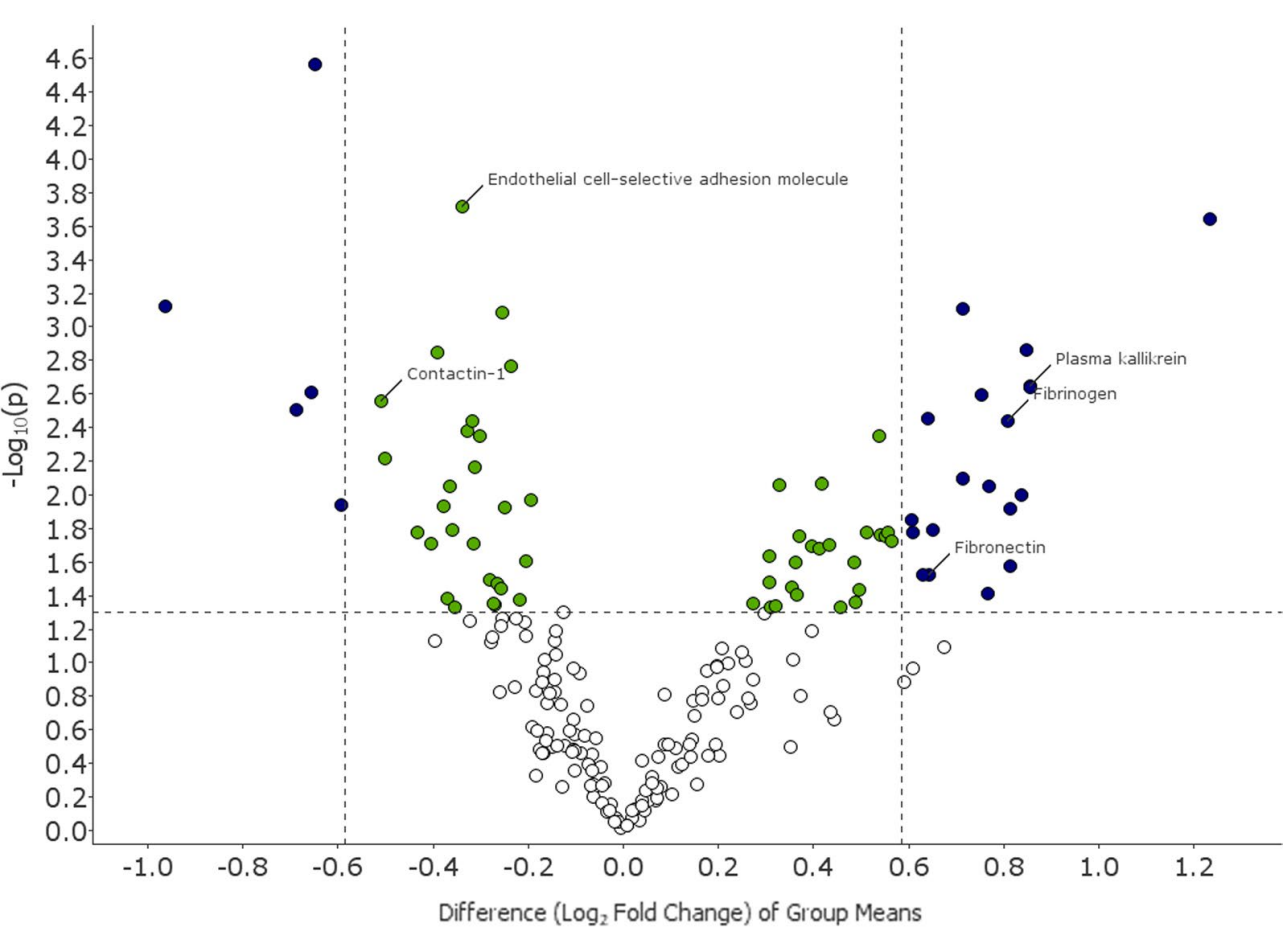
Volcano plot displaying differentially abundant proteins in subjects with non-productive cough. The most prominent proteins for separating those with non-productive cough and those without cough are shown in the top left and top right of the plot and are coloured blue. Proteins that differ between the groups to a smaller extent but have a significant p-value are coloured green. The negative log_10_ of the p-value is plotted on the y-axis and the difference (log_2_ fold change) on the x-axis, based on the t-test between the two groups adjusted for sex and the investigator

**Fig. 2:**
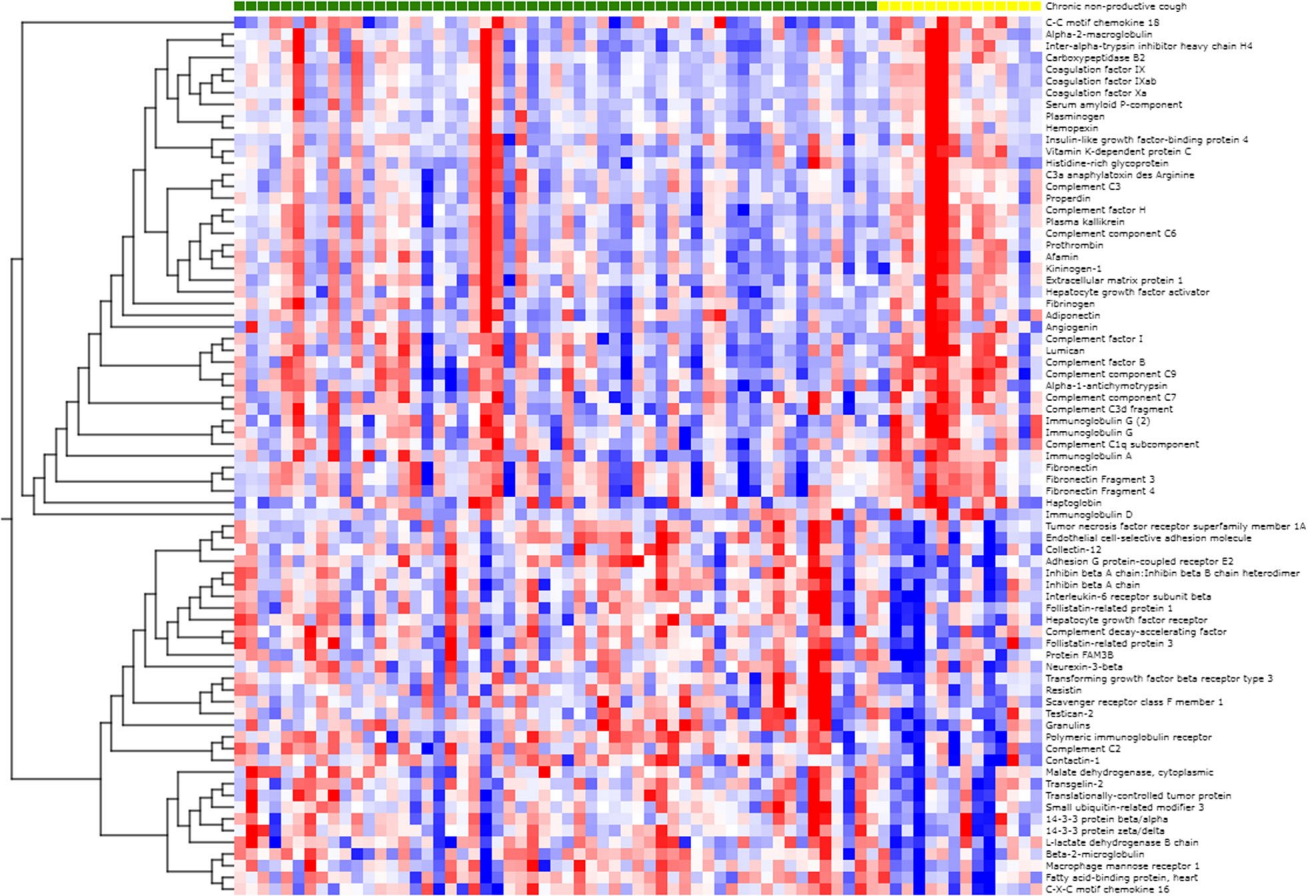
Clustering analysis of proteins based on the presence of non-productive cough. Proteins differentially abundant in those with non-productive cough (p < 0.05) were clustered by hierarchical clustering. The samples were ordered by the presence of non-productive cough

All in all, 203 proteins were detected in at least 50% of the 107 samples. Results from the analysis of the proteomics profile found 75 proteins significantly altered among participants with non-productive cough compared to controls, after adjusting for sex and investigator performing the PExA measurement (all with p-value < 0.05 and q-value ≤ 0.13, Table 2, Figs. 1 and 2). Thereof, 21 proteins had a q-value < 0.05. The five proteins with the most significant difference (p ≤ 0.0008, q ≤ 0.028) between participants with and without non-productive cough were adhesion G protein-coupled receptor E2 (fold change 0.64, p < 0.0001, q = 0.005), endothelial cell-selective adhesion molecule (ESAM) (fold change 0.79, p = 0.0002, q = 0.015), complement factor H (fold change 2.35, p = 0.0002, q = 0.015), polymeric immunoglobulin receptor (fold change 0.51, p = 0.0008, q = 0.028), and complement factor B (fold change 1.64, p = 0.0008, q = 0.028) (Table 2). The protein kallikrein was also significantly more common among participants with non-productive cough (fold change 1.81, p = 0.002, q = 0.04). Further details are seen in Table 2 and Fig. 1.

**Table 2:** Proteins differing between participants with non-productive cough and participants without cough (with p-value < 0.05)

Protein	Entrez Gene Symbol	p-value	q-value	Fold change
**Adhesion G protein-coupled receptor E2**	**ADGRE2**	**2.70E−05**	**0.005**	**0.64**
Endothelial cell-selective adhesion molecule	ESAM	1.90E**−**04	0.015	0.79
**Complement factor H**	**CFH**	**2.28E−04**	**0.015**	**2.35**
**Polymeric immunoglobulin receptor**	**PIGR**	**7.60E−04**	**0.028**	**0.51**
**Complement factor B**	**CFB**	**7.82E−04**	**0.028**	**1.64**
Inhibin beta A chain:Inhibin beta B chain heterodimer	INHBA, INHBB	8.19E**−**04	0.028	0.84
**Inter-alpha-trypsin inhibitor heavy chain H4**	**ITIH4**	**1.36E−03**	**0.036**	**1.80**
Inhibin beta A chain	INHBA	1.42E**−**03	0.036	0.76
Hepatocyte growth factor receptor	MET	1.72E**−**03	0.039	0.85
**Plasma kallikrein**	**KLKB1**	**2.25E−03**	**0.040**	**1.81**
**Prothrombin**	**F2**	**2.29E−03**	**0.040**	**1.81**
**Granulins**	**GRN**	**2.46E−03**	**0.040**	**0.63**
**Immunoglobulin A**	**IGHA1, IGHA2**	**2.54E−03**	**0.040**	**1.68**
Contactin-1	CNTN1	2.79E**−**03	0.040	0.70
**Fatty acid-binding protein, heart**	**FABP3**	**3.11E−03**	**0.041**	**0.62**
**Complement component C6**	**C6**	**3.51E−03**	**0.041**	**1.56**
Interleukin-6 receptor subunit beta	IL6ST	3.63E**−**03	0.041	0.80
**Fibrinogen**	**FGA, FGB, FGG**	**3.67E−03**	**0.041**	**1.75**
Complement C2	C2	4.16E**−**03	0.043	0.80
Vitamin K-dependent protein C	PROC	4.46E**−**03	0.043	1.45
kininogen-1	FSTL3	4.48E**−**03	0.043	0.81
Macrophage mannose receptor 1	MRC1	6.13E**−**03	0.057	0.71
Collectin-12	COLEC12	6.87E**−**03	0.061	0.81
**Complement C1q subcomponent**	**C1QA, C1QB, C1QC**	**8.02E−03**	**0.065**	**1.64**
Lumican	LUM	8.53E**−**03	0.065	1.33
Alpha-2-macroglobulin	A2M	8.76E**−**03	0.065	1.26
14–3-3 protein zeta/delta	YWHAZ	8.85E**−**03	0.065	0.78
**Coagulation factor IX**	**F9**	**8.94E−03**	**0.065**	**1.71**
**Plasminogen**	**PLG**	**9.96E−03**	**0.070**	**1.79**
Tumor necrosis factor receptor superfamily member 1A	TNFRSF1A	0.011	0.072	0.87
**C-X-C motif chemokine 16**	**CXCL16**	**0.011**	**0.072**	**0.66**
Beta-2-microglobulin	B2M	0.012	0.072	0.77
Small ubiquitin-related modifier 3	SUMO3	0.012	0.072	0.84
**Serum amyloid P-component**	**APCS**	**0.012**	**0.072**	**1.76**
**Hepatocyte growth factor activator**	**HGFAC**	**0.014**	**0.082**	**1.52**
**Immunoglobulin G**	**IGHG**	**0.016**	**0.082**	**1.57**
Resistin	RETN	0.016	0.082	0.78
**Hemopexin**	**HPX**	**0.017**	**0.082**	**1.53**
Immunoglobulin G (2)	IGHG	0.017	0.082	1.47
Fibronectin Fragment 3	FN1	0.017	0.082	1.42
Complement decay-accelerating factor	CD55	0.017	0.082	0.74
C3a anaphylatoxin des Arginine	C3	0.017	0.082	1.45
Carboxypeptidase B2	CPB2	0.018	0.082	1.46
Histidine-rich glycoprotein	HRG	0.018	0.082	1.29
Coagulation factor IXab	F9	0.019	0.084	1.48
Protein FAM3B	FAM3B	0.019	0.084	0.80
Transgelin-2	TAGLN2	0.020	0.084	0.76
Angiogenin	ANG	0.020	0.084	1.35
Complement component C9	C9	0.020	0.084	1.32
Afamin	AFM	0.021	0.085	1.33
Complement factor I	CFI	0.023	0.092	1.24
Transforming growth factor beta receptor type 3	TGFBR3	0.025	0.096	0.87
Fibronectin Fragment 4	FN1	0.025	0.096	1.29
Properdin	CFP	0.025	0.096	1.40
**Coagulation factor Xa**	**F10**	**0.027**	**0.099**	**1.76**
**Kininogen-1**	**KNG1**	**0.030**	**0.107**	**1.55**
**Fibronectin**	**FN1**	**0.030**	**0.107**	**1.56**
14–3-3 protein beta/alpha	YWHAB	0.032	0.112	0.82
Complement C3d fragment	C3	0.033	0.114	1.24
Translationally-controlled tumor protein	TPT1	0.034	0.115	0.83
Complement C3	C3	0.035	0.117	1.28
Follistatin-related protein 1	FSTL1	0.036	0.118	0.84
C–C motif chemokine 18	CCL18	0.037	0.118	1.41
**Haptoglobin**	**HP**	**0.039**	**0.122**	**1.70**
Alpha-1-antichymotrypsin	SERPINA3	0.039	0.122	1.29
Malate dehydrogenase, cytoplasmic	MDH1	0.042	0.126	0.77
Scavenger receptor class F member 1	SCARF1	0.042	0.126	0.86
Adiponectin	ADIPOQ	0.044	0.126	1.40
Testican-2	SPOCK2	0.044	0.126	0.83
Complement component C7	C7	0.044	0.126	1.21
Neurexin-3-beta	NRXN3	0.045	0.126	0.83
Immunoglobulin D	IGHD	0.046	0.126	1.25
Extracellular matrix protein 1	ECM1	0.046	0.126	1.37
Insulin-like growth factor-binding protein 4	IGFBP4	0.047	0.126	1.24
L-lactate dehydrogenase B chain	LDHB	0.047	0.126	0.78

Proteins in bold have a fold change above 1.5 or below 0.7

Additional 6 proteins were identified above the LOD among more than half of those with non-productive cough, but in fewer subjects without cough. The proteins Tenascin-C, immunoglobulin M, and Neurogenic locus notch homolog protein 3 (NOTCH3) had a difference of more than 20 percentage points. Further details are given in Table 3.

**Table 3:** Proteins in PEx more frequently above LOD among those with non-productive cough

Protein	Entrez Gene Symbol	Subjects with chronic non-productive cough	Subjects without chronic non-productive cough
		% of samples with RFU value > LOD	% of samples with RFU value > LOD
Neurogenic locus notch homolog protein 3	NOTCH3	50.0	21.8
Immunoglobulin M	IGHM	64.3	38.2
Tenascin-C	TNC	71.4	45.5
Metalloproteinase inhibitor 1		64	45
Tryptase beta-2		64	45
Mast/stem cell growth factor receptor Kit		57	45

Proteins identified above the LOD among more than half of those with non-productive cough, but among less than half of those without cough

A sensitivity analysis including current smokers, with adjustment for smoking status, found no significant differences in the findings described above (results not shown).

### STRING analysis results

**Fig. 3:**
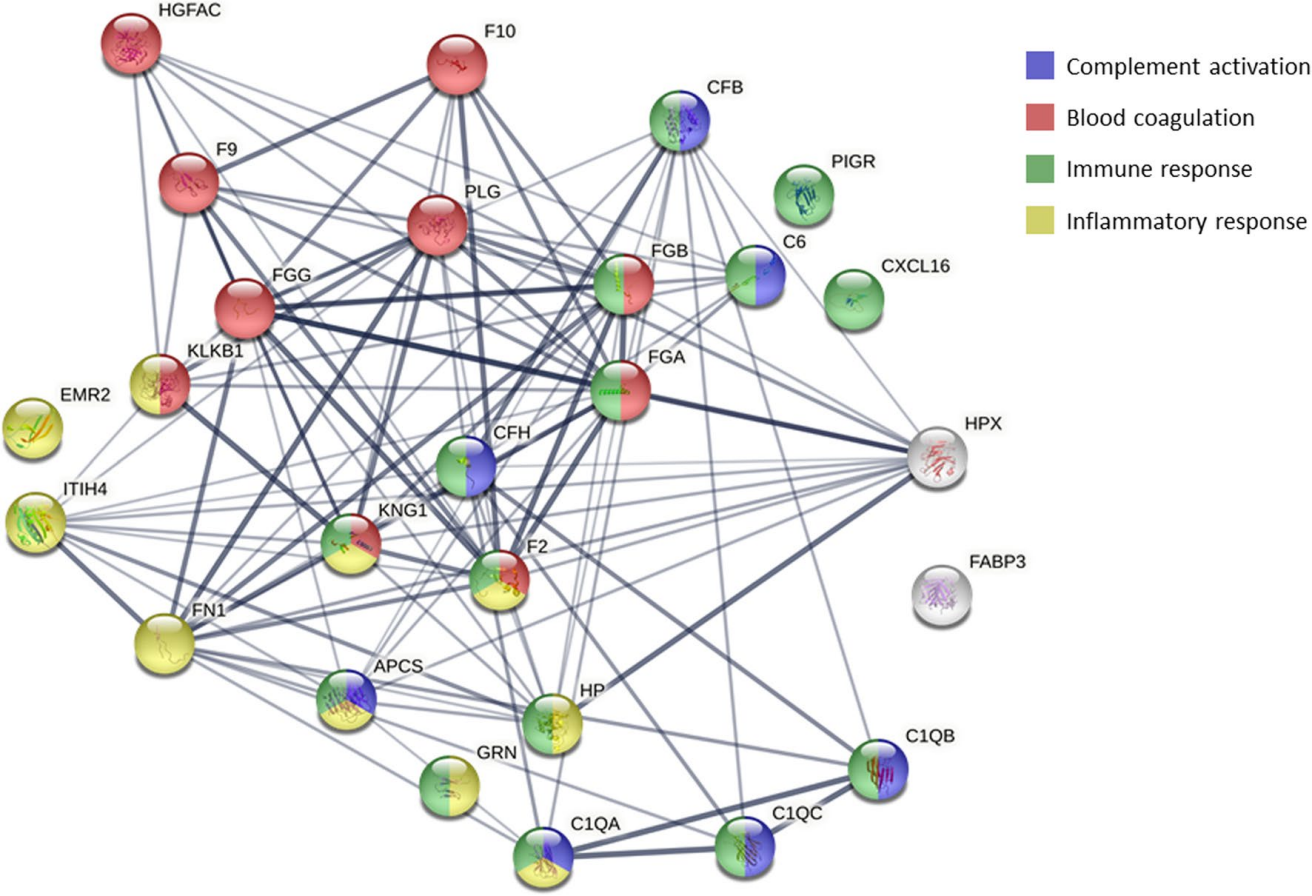
Protein interaction analysis in STRING using differentially abundant proteins in non-productive cough

The STRING analysis identified mostly proteins associated with pathways regulating the immune and inflammatory responses, as well as with complement activation and coagulation (Fig. 3).

## Discussion

In this first explorative study on exhaled biomarkers from peripheral airways of individuals with a non-productive cough, we found through proteomics analysis that 75 proteins were significantly altered, compared to individuals without cough. The magnitude of associations, and the common pathways involved for the identified proteins, suggest that these findings represent true associations between altered biochemical processes in the distal airways and non-productive cough.

Various immune system pathways have been suggested to be involved in the pathogenesis of cough, both eosinophilic (Th2 inflammation), neutrophilic, and lymphocytic [12], suggesting that there are different pathological mechanisms involved and that chronic cough is a heterogenous disease. Currently, it is unclear which of these pathways are most important, or if they mostly associate with certain subgroups of patients with chronic cough. Certain biomarkers may be related to the phenotype of cough, when identifiable. Indeed, a previous study found substance P to be specifically elevated in exhaled breath condensate of patients with gastroesophageal reflux and cough [11], and patients with chronic cough and elevated fractional exhaled nitric oxide (FeNO, reflecting eosinophilic inflammation) respond better to inhaled corticosteroids than those with low FeNO [15].

We did not identify any study on airway proteomics in chronic cough. In our data, we found Tenascin-C, a biomarker associated with chronic inflammation [26], to be more commonly identified in PEx among participants with non-productive cough, and Contactin-1 to be elevated in PEx. Contactin-1 is implicated in asthma pathogenesis, as a signal molecule in airway epithelium-derived exosomes, inducing a Th2 inflammation [27]. Thus, we found some support for a role of Th2 inflammation in chronic cough.

We also found NOTCH3 to be more often identified above the limit of detection in PEx samples among participants with cough compared to those without (50% vs 22%, respectively). NOTCH3 is centrally involved in epithelial homeostasis and regeneration [28], but its role in chronic cough has not been studied.

The complement system, a part of the innate immune system, and the coagulation system are known to crosstalk [29]. Both the coagulation- and complement systems are also known to be affected in some airway diseases, such as asthma [30]. In our study, we found numerous proteins involved in both the complement and coagulation systems to be affected, suggesting a role for the innate immune system in non-productive cough. Indeed, as cough is essentially a first-line mechanism to clear debris from the airways, it is perhaps not surprising that the innate immune system—a first-line immune response system—may affect the cough reflex.

The complement system has not been extensively studied in chronic cough. In one study on COPD patients, the complement factors C3 and C4 were found to be lower in serum among those with cough and expectoration [31]. We did not identify other studies on chronic cough and complement activation. Further studies are needed to further explore the role of the complement and coagulation systems in cough.

Fibrinogen was found to be increased, a protein which is both implicated in formation of extracellular matrix, and involved in inflammation and coagulation in the blood [32, 33]. Fibrinogen has previously been described to be increased in blood samples among patients with asthma and cough, compared to patients with asthma but without cough [32]. Also, the extracellular matrix protein fibronectin was increased among those with non-productive cough, a protein that also has been implicated in the pathogenesis of lung fibrosis [34, 35].

Some proteins identified deserve specific attention with regard to the cough hypersensitivity theory. For example, plasma kallikrein, which we found to be elevated, is a protein that cleaves kininogen to form bradykinin. Bradykinin is well known to be implicated in chronic cough [36]. Other studies have also indicated this possible association, where plasma kallikrein may be implicated in inducing cough through activating bradykinins [37, 38]. Furthermore, the kallikrein-kinin system (including bradykinin) has been implicated to play a role in neuroinflammation, both centrally as in Alzheimer's disease, as well as affect the peripheral nervous system. Our data therefore support the cough hypersensitivity theory as a plausible mechanism in chronic cough.

Another interesting aspect is the potential impairment of tight junctions, which may lead to increased permeability and susceptibility of airway nerve receptors to external stimuli [8, 9]. In our data, the protein ESAM, which contributes to the integrity of tight junctions [39], was significantly lowered in PEx samples of participants with non-productive cough. Unfortunately, many of the tight junction proteins analysed in the SOMAscan platform were below the detection limit in the present study. We did not identify other studies directly addressing epithelial permeability in chronic cough, but the issue has been discussed in asthma, where increased epithelial permeability is suggested to be a part of the pathogenesis [8].

### Strengths and weaknesses

Some of the novelties and main strengths of this study are the combination of a non-invasive collection of non-diluted biosamples from the small airways, and the unbiased proteomic approach. However, some methodological issues need to be discussed. First of all, the sample size is small and an independent validation cohort is needed to confirm our findings. We also observed variability in the protein-profile depending on the investigator performing the PEx sampling, possibly explained by differences in how the breathing maneuver was performed, and as a result the statistical analysis had to be adjusted accordingly. Choosing to include proteins in the analysis with RFU values > LOD in more than 50% of samples could be considered another possible limitation. However, due to the exploratory nature of the study, we chose to select 50% as the limit instead of other commonly used percentages such as 70–80%, to open the analytical window and explore proteins that could potentially be missed otherwise. For that same reason and also due to the small sample size, a more inclusive approach was chosen and differences in protein abundance were considered significant at p-value < 0.05.

Some potentially interesting biomarkers, such as the neuroinflammatory marker Substance P which is implicated in cough [12], were not among the proteins analysed by the SOMAscan platform. Also, in part because of the relatively low sample volume, some proteins were not easily identified, such as Cadherin-1 which was only detected above LOD in 6% of all samples.

Because of the exploratory nature of this study, strong conclusions cannot be made, even though the results show interesting findings. The clear differences in abundance of many proteins between participants with non-productive cough and those without cough, merit larger studies to validate these findings, preferably including clinical patients with chronic cough.

## Conclusions

In this exploratory study on the proteomic profile in distal airway samples from individuals with non-productive cough, we found significant associations between non-productive cough and altered abundance of 75 proteins, in biological samples that originate from distal airways. Interestingly, many of these proteins are involved in pathways regulating the immune and inflammatory responses, as well as complement activation, coagulation, neuroinflammation, and epithelial junction integrity. Further studies are needed to validate these findings, and to explore the role of these altered pathways in the pathology of cough.

## Data Availability

The datasets generated and/or analysed during the current study are not publicly available as primary data analysis is still ongoing for other research questions, but are available from the corresponding author on reasonable request.

## Abbreviations

PEx: Particles in exhaled air (samples)
PExA: Particles in exhaled air (method / equipment)
GLP: Good laboratory practice
RFU: Relative fluorescent unit
LOD: Limit of detection
FVC: Forced vital capacity
FEV1: Forced expired volume in one second
TNF: Tumour necrosis factor
IOS: Impulse oscillometry
FeNO: Fractional exhaled nitric oxide
R5: Resistance at 5 Hz
R20: Resistance at 20 Hz
AX: Area under the reactance curve
STRING: Search Tool for the Retrieval of INteracting Genes/proteins
NOTCH3: Neurogenic locus notch homolog protein 3
ESAM: Endothelial cell-selective adhesion molecule
